# Prevalence of restless legs syndrome during the 2019 coronavirus disease pandemic in South Korea: A nationwide cross-sectional population-based study

**DOI:** 10.3389/fneur.2022.1101711

**Published:** 2022-12-22

**Authors:** Jiyoung Kim, Kyung Wook Kang, Keun Tae Kim, Yong Won Cho

**Affiliations:** ^1^Department of Neurology and Sleep Disorder Center, Bio Medical Research Institute, Pusan National University Hospital, Pusan National University School of Medicine, Busan, South Korea; ^2^Department of Neurology, Chonnam National University Hospital, Chonnam National University School of Medicine, Gwangju, South Korea; ^3^Department of Neurology, Keimyung University School of Medicine, Daegu, South Korea

**Keywords:** restless legs syndrome, chronic persistent restless legs syndrome, COVID-19, COVID-19 vaccines, epidemiology

## Abstract

**Background:**

The 2019 coronavirus disease (COVID-19) pandemic has been associated with a significant increase in sleep disorders. This study aimed to determine the prevalence of restless leg syndrome (RLS) and the effect of COVID-19 on RLS during the pandemic in Korea.

**Methods:**

The National Sleep Survey of South Korea 2022 was employed in this study. This study was a large population-based web survey using a structural questionnaire of a four thousand representative sample of individuals aged 20–69 years in Korea. The survey was conducted between January 2022 and February 2022 during the COVID-19 pandemic. RLS was diagnosed using the Korean version of the paradigm of questions for epidemiological studies of RLS. Chronic persistent RLS was defined for individuals with RLS symptoms at least twice a week.

**Results:**

Six hundred forty-nine (16.2%) and 172 (4.3%) patients were classified as having RLS and chronic persistent RLS, respectively. Female sex, being employed, the presence of COVID-19 vaccine-related adverse events, decreased sleep duration, the presence of EDS, and current treatment for insomnia were significantly associated with chronic persistent RLS.

**Conclusion:**

During the COVID-19 pandemic, the prevalence of RLS and chronic persistent RLS in the adult Korean population was higher than that reported in previous studies.

## 1. Introduction

Restless legs syndrome (RLS), also known as Willis-Ekbom disease, is a common sensorimotor neurological disorder characterized by core clinical symptoms, such as the urge to move, usually accompanied by an uncomfortable sensation. It typically occurs during periods of rest or inactivity, is relieved by movement, and begins or worsens at night (1). The prevalence of RLS ranges from 3.9 to 14.3% worldwide based on four minimal criteria from the 2003 international restless legs syndrome study group (IRLSSG) (1, 2). The prevalence is higher in women than in men and increases with age (3). Furthermore, the prevalence of RLS differs depending on the region; North America and Europe tend to have a higher prevalence than Asia and Africa (4).

A recent international collaborative study using a harmonized questionnaire [items on sleep, wake and dreaming, physical health, mental health, and coronavirus disease-19 (COVID-19)] revealed a higher prevalence of insomnia, anxiety, and depression during the COVID-19 pandemic than during non-pandemic times (5). Considering that RLS is closely related to insomnia, depression, and anxiety (6, 7), it is also necessary to suppose that the COVID-19 pandemic affected the prevalence of RLS. In a cross-sectional survey of women with long COVID-19 at both current and pre-COVID time points, the prevalence of RLS also increased after COVID-19 compared with before COVID-19 (8). Furthermore, a longitudinal observational study reported increased RLS severity during the early period of the COVID-19 pandemic (9).

We hypothesized that the prevalence of RLS increased in the general population during the COVID-19 pandemic. To test this, we determined the RLS prevalence during the COVID-19 pandemic and compared it with our previous study conducted before the pandemic (6).

## 2. Methods

### 2.1. Study population and survey process

This study was conducted as part of the National Sleep Survey of South Korea 2022, which is a nationwide population-based web-based survey. The survey was conducted on individuals aged 20–69 and was performed from January to February 2022, during the COVID-19 pandemic. In 2021, the number of individuals aged 20–69 in South Korea was 37,133,171, and the minimum sample size was 3,998 when set as a sampling error of ± 1.55 points within the 95% confidence interval (CI). Therefore, the final number of participants in this study was 4,000 individuals. All participants of this study were part of the panels of an online survey service provider: Embrain Public Company. It maintains a panel of more than 1.5 million individuals. The panels were gathered voluntarily and consisted of individuals who could participate in the online survey. This study data was collected from the panels by using a proportional stratified sampling method according to sex, age groups and place of residence to generate a national representation of adult individuals aged 20–69 from South Korea. This survey was performed by the Embrain Public Company, which was launched by the Epidemiology Committee of the Korean Sleep Research Society. The study was conducted in accordance with the Declaration of Helsinki and approved by the institutional review board of Keimyung University Hospital (IRB No. DSMC 2021-12-063). All the participants provided written informed consent.

### 2.2. RLS assessment

RLS was assessed using the Korean-validated version of the paradigm of questions for epidemiological studies of RLS, which consisted of the following three questions regarding RLS symptoms for diagnosis and the frequency of symptoms for severity (10): (1) an urge to move the legs, usually associated with unpleasant sensations, (2) symptoms occurring during periods of rest and symptoms relieved by movement, and (3) symptoms worse in the evening or night. It has been successfully translated and validated into Korean. The sensitivity and specificity of the paradigm of questions for epidemiological studies of RLS were 79–85 and 94–96% in the validated study which was conducted by Cho et al. (10). If the participants had all three symptoms, they were classified as having RLS. We thought that participants who answered “yes” to all three questions met the four essential criteria of the IRLSSG (1). In addition, the frequency of RLS symptoms in participants with RLS was evaluated. Among participants with RLS, chronic persistent RLS was defined as RLS symptoms experienced at least twice a week (1).

### 2.3. The COVID-19 questionnaires

The questions related to COVID-19 included the experience of being diagnosed with COVID-19, COVID-19 vaccination history (more than twice), and the presence of vaccine-related adverse events in those who received COVID-19 vaccination more than twice. COVID-19 vaccine-related adverse events (CAEs) included all side effects experienced immediately after COVID-19 vaccination; however, the severity of the adverse events was not evaluated in this study. To avoid information bias about COVID-19, the main purpose of the study was introduced to participants as a sleep health survey.

### 2.4. Sleep parameter and other variables

We investigated subjective sleep-related variables, such as sleep onset latency (SOL), sleep duration, presence of excessive daytime sleepiness (EDS), and current treatment for insomnia. Self-reported sleep duration and SOL were calculated using the answers to the questionnaire, which were asked during weekdays and weekends separately. In order to reduce recall bias, we asked the participants their sleep duration and SOL of the last month. Sleep duration and SOL were calculated as the average values on weekdays and weekends: (weekday value × 5) + (weekend value × 2)/7. In our analyses, we set the SOL threshold at 21 min and the sleep duration threshold at 7 h, which are known to be associated with poor sleep (11). The optimal cutoff value of SOL for discrimination of insomnia sufferers from normal sleepers has been suggested to be 20 min or longer in a recent study using standardized sleep interviews as well as polysomnography (12). We evaluated EDS using the Epworth Sleepiness Scale (ESS), which has been validated in Korea (13). Participants with ESS scores ≥11 were classified as having EDS. Heavy alcohol consumption was defined according to the Korean Alcohol Guidelines for Primary Care Physicians (14).

### 2.5. Statistical analysis

Descriptive statistics were performed for demographic- and RLS-related variables. The Shapiro-Wilk test was used to evaluate the normality of the distribution in a continuous variable. After normality was confirmed, the Student's *t-*test was used to compare continuous variables. A chi-square test was performed to determine differences in categorical variables. The tendency of the prevalence of chronic persistent RLS by age was evaluated using a linear-by-linear association. Multivariate logistic regression analysis was performed to assess the independent contribution of the factors that influenced the presence of chronic persistent RLS. The results are expressed as odds ratios (ORs) with 95% CIs. A two-sided *P* < 0.05 was considered statistically significant. All statistical analyses were performed using SPSS version 22 (IBM Corp., Armonk, NY, USA).

## 3. Results

### 3.1. Study population

The mean age of the participants was 44.8 ± 13.3 years. Among the participants, 1,965 (49.1%) and 2,035 (50.9%) were women and men, respectively. Table 1 presents the demographic characteristics of the participants. Of them, 1.2% (48) had a history of COVID-19 infection, 92.6% (3,704) received two or more vaccinations, and 38.2% (1,529) experienced CAE.

**Table 1:** Basic demographics and COVID-19-related characteristics of the participants.

	**Participants (*N* = 4,000)**	**RLS (+)** **(*N* = 649)**	**RLS (-) (*N* = 3,351)**	*P* ^*^
Age, years	44.8 ± 13.3	48.6 ± 12.7	44.1 ± 13.3	<0.001
Sex				<0.001
Male	2,035 (50.9%)	268 (41.3%)	1,767 (52.7%)	
Female	1,965 (49.1%)	381 (58.7%)	1,584 (47.3%)	
Region				0.476
Rural	2,211 (55.3%)	367 (56.5%)	1,844 (55%)	
Urban	1,789 (44.7%)	282 (43.5%)	1,507 (45%)	
Marital status				<0.001
Current married	2,338 (58.5%)	454 (70.0%)	1,884 (56.2%)	
Current unmarried	1,662 (41.6%)	195 (30.0%)	1,467 (43.8%)	
State of employment				0.035
Employed	2,852 (71.3%)	485 (74.7%)	2,367 (70.6%)	
Unemployed	1,148(28.7%)	164 (25.3%)	984 (29.4%)	
Alcohol				
Alcohol consumption (g/week)	49.93 ± 83.42	49.84 ± 81.07	49.94 ± 83.88	0.976
Heavy alcohol drinking	638 (16.0%)	111 (17.1%)	527 (15.7%)	0.381
Smoking	1,862 (46.6%)	295 (45.5%)	1,567 (46.8%)	0.541
BMI, kg/m^2^	23.71 ± 3.67	23.76 ± 3.73	23.70 ± 3.66	0.724
Hypertension	669 (16.7%)	135 (20.8%)	534 (15.9%)	0.002
COVID-19				
Infection history (+)	48 (1.2%)	12 (1.8%)	36 (1.1%)	0.097
Vaccination more than 2 times	3,704 (92.6%)	615 (94.8%)	3,089 (92.2%)	0.022
CAE (+)	1,529 (38.2%)	271 (44.1%)	1,258 (37.5%)	0.124
Poor sleep				
Sleep duration ≥7 h	2,009 (50.2%)	296 (45.6%)	1,713 (51.1%)	0.01
SOL≥21 min	2,307 (57.7%)	427 (65.8%)	1,880 (56.1%)	<0.001
EDS	510 (12.8%)	125 (19.3%)	385 (11.5%)	<0.001
Person currently being treated for insomnia	87 (2.2%)	28 (4.3%)	59 (1.8%)	<0.001

* Comparison between participants with and without RLS.

RLS, restless legs syndrome; COVID-19, coronavirus disease-19; BMI body max index; CAE, COVID-19 vaccine-related adverse events; SOL, sleep onset latency; EDS, excessive daytime sleepiness.

### 3.2. Prevalence and clinical characteristics of RLS

Of the 4,000 participants, 649 (16.2%) were classified as having RLS. The prevalence of RLS was significantly higher in women than in men (19.4 vs. 13.2%, *p* < *0.001*). Participants with RLS received more COVID-19 vaccinations and had significantly poorer sleep habits than those without RLS (Table 1).

### 3.3. Prevalence and clinical characteristics of chronic persistent RLS

**Figure 1:**
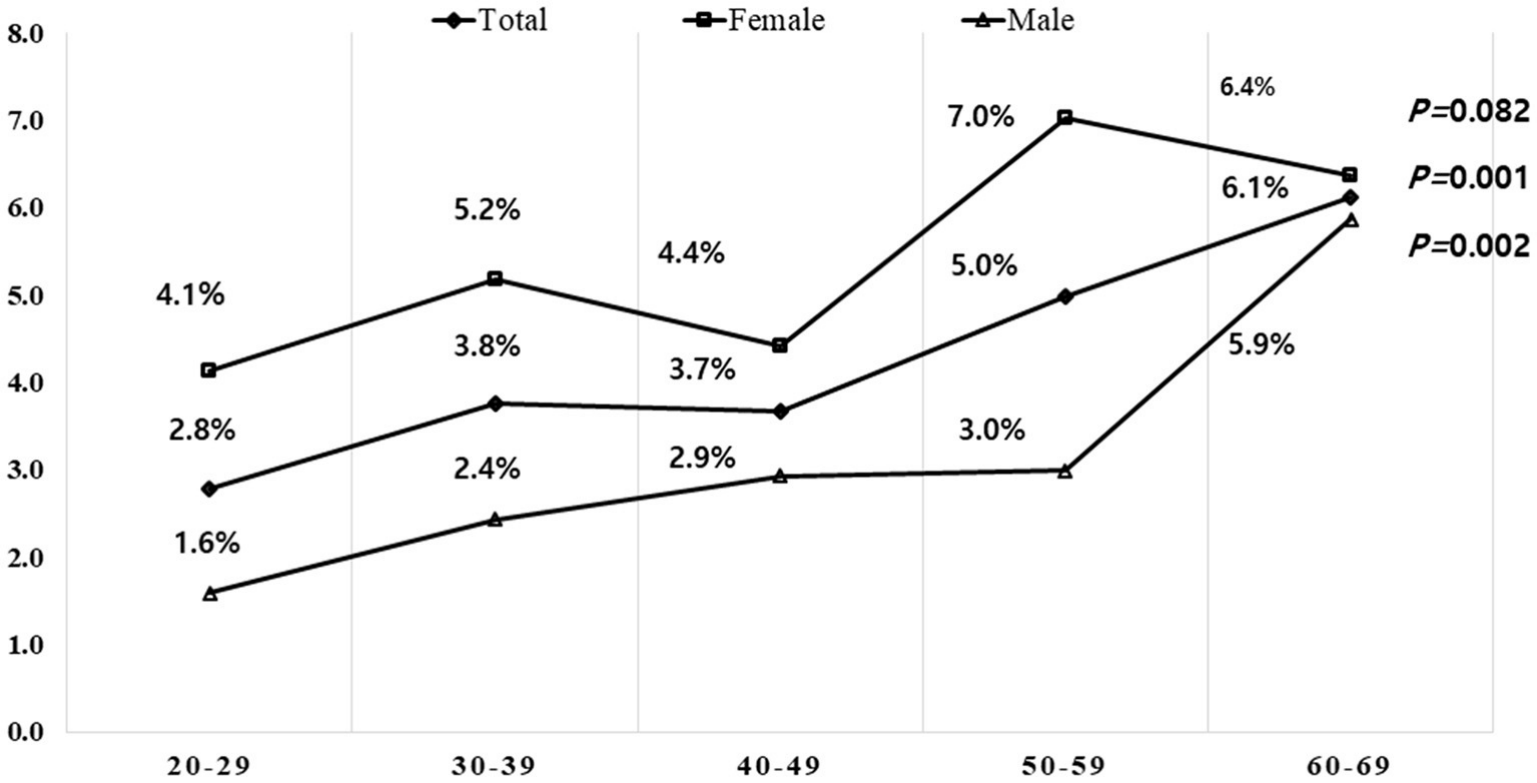
Prevalence of chronic persistent RLS by age and sex. The prevalence was higher in females than males in all age groups. The prevalence of chronic persistent RLS increased with age in males but not significantly with age in females. Statistical analysis was performed using linear by linear association. RLS, restless legs syndrome.

Of the 4,000 participants, 172 (4.3%) were classified as having chronic persistent RLS. Women showed a higher tendency than men in all age groups (Figure 1). As age increased, the prevalence of chronic persistent RLS increased significantly in the total (*p* = *0.001*) and male (*p* = *0.002*) groups but not in the female group (*p* = *0.082*). In the comparison between participants with chronic persistent RLS and those without RLS, the mean age of the participants and prevalence of women with chronic persistent RLS were significantly higher than those without RLS. Participants with chronic persistent RLS had a higher prevalence of hypertension than those without RLS. Furthermore, patients with chronic persistent RLS had significantly higher rates of COVID-19 infection and CAE than those without RLS. Most sleep parameters were significantly worse in participants with chronic persistent RLS than in those without RLS (Table 2).

**Table 2:** Basic demographics and COVID-19-related characteristics of participants with and without chronic persistent RLS.

	**Chronic persistent RLS (+) (*N* = 172)**	**RLS (-)** **(*N* = 3,351)**	*P* ^*^
Age, (years)	48.0 ± 13.4	44.1 ± 13.3	<0.001
Sex			<0.001
Male	64 (37.2%)	1,767 (52.7%)	
Female	108 (62.8%)	1,584 (47.3%)	
Region			0.090
Rural	106 (61.6%)	1,844 (55.0%)	
Urban	66 (38.4%)	1,507 (45.0%)	
Marital status			0.001
Current married	118 (68.6%)	1,884 (56.2%)	
Current unmarried	54 (31.4%)	1,467 (43.8%)	
State of employment			0.040
Employed	134 (77.9%)	2,367 (70.6%)	
Unemployed	38 (22.1%)	984 (29.4%)	
Alcohol			
Alcohol consumption (g/week)	55.6 ± 89.2	49.94 ± 83.88	0.388
Heavy alcohol drinking	34 (19.8%)	527 (15.7%)	0.158
Smoking	80 (46.5%)	1,567 (46.8%)	0.949
BMI, kg/m^2^	23.8 ± 3.6	23.70 ± 3.66	0.713
Hypertension	41 (23.8%)	534 (15.9%)	0.006
COVID-19			
Infection history (+)	5 (2.9%)	36 (1.1%)	0.029
Vaccination more than 2 times	163 (94.8%)	3,089 (92.2%)	0.214
CAE (+)	83 (50.9%)	1,258 (37.5%)	0.010
Poor sleep			
Sleep duration ≥7 h	60 (34.9%)	1,713 (51.1%)	<0.001
SOL≥21 min	116 (67.4%)	1,880 (56.1%)	0.003
EDS	37 (21.5%)	385 (11.5%)	<0.001
Person currently being treated for insomnia	13 (7.6%)	59 (1.8%)	<0.001

* Comparison between participants with chronic persistent RLS and healthy controls without RLS.

RLS, restless legs syndrome; COVID-19, coronavirus disease-19; BMI body max index; CAE, COVID-19 vaccine-related adverse events; SOL, sleep onset latency; EDS, excessive daytime sleepiness.

Multivariate logistic regression analysis was performed to investigate factors associated with an increased risk of chronic persistent RLS. Age, sex (female), employment status, hypertension, and CAE were significantly associated with chronic persistent RLS in Model 1. In Model 2, sleep parameters were added to the covariates in Model 1, and CAE was found to be significantly associated with an increased risk of chronic persistent RLS (Table 3).

**Table 3:** Multivariate logistic regression analysis of chronic persistent RLS.

	**OR (95% CI)**			
	**Model 1**	***p***	**Model 2**	***p***
Age, years	1.020 (1.007–1.034)	0.003	1.014 (0.998–1.030)	0.081
Female	2.066 (1.459–2.926)	<0.001	2.170 (1.519–3.099)	<0.001
Marital status (married)	1.340 (0.909–1.975)	0.143	1.392 (0.937–2.067)	0.101
State of employment (employed)	1.675 (1.134–2.474)	0.010	1.669 (1.121–2.486)	0.012
Heavy alcohol dinking	1.276 (0.841–1.936)	0.168	1.252 (0.822–1.907)	0.240
Smoking	1.210 (0.817–1.790)	0.238	1.131 (0.761–1.680)	0.403
BMI, kg/m^2^	1.025 (0.979–1.072)	0.303	1.024 (0.978–1.072)	0.330
Hypertension	1.542 (1.014–2.343)	0.043	1.441 (0.942–2.205)	0.092
CAE (+)	1.482 (1.071–2.052)	0.018	1.424 (1.024–1.980)	0.036
Sleep duration ≥7 h			0.633 (0.453–0.885)	0.007
SOL≥21 min			1.357 (0.964–1.911)	0.080
EDS			2.283 (1.533–3.402)	<0.001
Person currently being treated for insomnia			3.989 (2.052–7.752)	<0.001

Model 1, adjusted for sociodemographic factors (age, sex, marital status, employment, alcohol consumption, smoking, BMI), hypertension, and CAE; Model 2, adjusted for sociodemographic factors, hypertension, CAE, and sleep parameters (sleep duration, SOL, EDS, insomnia).

RLS, restless legs syndrome; OR, odds ratio; CI, confidence interval; BMI, body mass index; CAE, COVID-19 vaccine-related adverse events; SOL, sleep onset latency; EDS, excessive daytime sleepiness.

## 4. Discussion

Our study is an epidemiologic study of RLS in a large Korean adult population performed during the COVID-19 pandemic. In this cross-sectional survey, we found that the prevalence of RLS (16.2%) and chronic persistent RLS (4.3%) was higher than that in the 2007 survey before COVID-19 (6). A previous national general population-based study reported that the prevalence of RLS, which met all four essential criteria, and chronic persistent RLS was 3.9% (*n* = 194) and 1.7% (*n* = 83), respectively, in a telephone interview survey using the Korean version of the Johns Hopkins telephone diagnostic interview in Korea (6). In another cross-sectional general population-based epidemiological study using four essential criteria of the IRLSSG 2003 to diagnose RLS, the prevalence of RLS was 5.5, and 2.5% had RLS symptoms at least once a week in 2009 (15). It is necessary to determine which factors cause inconsistencies compared with previous studies. We believe that the higher prevalence in the present study could be related to many unprecedented changes during the COVID-19 pandemic.

Since the participants in the current study had a low rate of COVID-19 infection (1.2%, 48/40,000), it was limited to evaluating the association between RLS development and COVID-19 infection. However, a hypothesis has been raised about the possibility of an association between RLS development and the postinfectious immune response after COVID-19 (16). A cross-sectional survey that investigated the change in RLS prevalence pre- and post-COVID-19 in women with long-COVID symptoms reported an increased prevalence of RLS from 5.7 to 14.8% before and after long COVID symptoms, respectively (8). The possible relationship between increased RLS prevalence and the COVID-19 vaccination may also be considered. One study prospectively studied 628 adults and found that 7.0% of the participants reported symptoms suggestive of RLS after COVID vaccination (17). Although the information is very limited and further research is still needed, the development of RLS in relation to COVID-19 vaccination can be considered to cause acute immune reactions, local inflammation, and somatic symptoms (18). Other factors that increase the prevalence of RLS should be considered during the COVID-19 pandemic. The prevalence of insomnia has been reported to be higher than that in the prepandemic era in general population studies as well as in meta-analyses since the COVID-19 pandemic (19, 20). In our dataset, 58% of the participants had a SOL of 21 min or longer. Interestingly, in international studies performed during the COVID-19 pandemic, psychological problems such as anxiety and depression were significantly associated with insomnia and were also very prevalent compared with the prepandemic periods (5). Thus, during the pandemic, increased anxiety and depression are related to the increasing prevalence of RLS, which is known for its positive correlation with psychological problems, although no apparent causal relationship is known (21, 22). In our study, chronic persistent RLS was significantly associated with CAE and sleep variables such as sleep duration, EDS, and insomnia, which are known to be associated with anxiety (19, 23). During the COVID-19 pandemic, prevaccine-related side effect expectations, worry about COVID-19, and depression could have predicted CAE in a prospective longitudinal study (24). Although there was no significant difference in the rate of CAE between participants with and without RLS, participants with chronic persistent RLS reported more CAE. These findings could be explained that psychological problems were enough to cause a CAE is more related to RLS with a frequency ≥2/week than <2/week. In other words, as the severity and frequency of RLS is associated with the severity of depression and anxiety (25, 26), chronic persistent RLS may have a higher level of depression and anxiety than intermittent RLS. In our study, the increased anxiety and depression during the pandemic era may affect both the association between CAE and chronic persistent RLS and the increased prevalence of RLS compared with prepandemic era. Taken together, based on our study, we speculate that postinfectious immune responses regarding COVID-19 infection, COVID-19 vaccination, and negative psychological aspects, such as anxiety and depressed mood during the pandemic, may have affected the increased prevalence of RLS and chronic persistent RLS.

To the best of our knowledge, this study is the first to investigate the prevalence of RLS in the general population during the COVID-19 pandemic in Korea. The strengths of the present study include the large sample size and inclusion of multiple potential confounders, such as sleep parameters, alcohol habits, hypertension, and the use of a structured questionnaire. This study had some limitations. First, we defined RLS based on diagnostic questionnaires and RLS mimics, such as leg cramps, arthritis, neuropathy, and venous stasis, which might have been misclassified as RLS. However, this is an inevitable limitation that might have also been included in pre-COVID population-based studies. Therefore, it could not be a barrier to our finding that the prevalence of RLS and chronic persistent RLS during the COVID-19 pandemic was higher than pre-COVID. Second, as detailed anxiety and depression were not investigated in the present study, the effect of the state of psychological symptoms on RLS could not be evaluated in detail during the COVID-19 pandemic. Third, the increase in the prevalence of RLS may have been affected by other factors that were not investigated in this study. Compared with the past, the evaluation and diagnosis of RLS have become more common, and information about RLS has spread, and these factors can increase the prevalence of RLS. However, this study surveyed the prevalence of participants in the general population who met the RLS diagnostic criteria of validated questionnaires in South Korean adults. Therefore, it is unlikely that the active evaluation and diagnosis of RLS affected the prevalence of this study.

During the COVID-19 pandemic, the prevalence of RLS and chronic persistent RLS showed a higher prevalence compared with results from previous studies pre-COVID. We believe that many COVID-19-related contributing factors might have increased the prevalence of RLS during the pandemic. Further research is needed to explore the relationship between RLS and COVID-19. To clarify the relationship between COVID-19 and RLS, it is also necessary to re-evaluate the change in the prevalence of RLS after the end of the COVID-19 pandemic.

## Data availability statement

The raw data supporting the conclusions of this article will be made available by the authors, without undue reservation.

## Ethics statement

The studies involving human participants were reviewed and approved by the Institutional Review Board of Keimyung University Hospital. The patients/participants provided their written informed consent to participate in this study.

## Author contributions

JK and KWK: data curation, analysis, and writing original draft. YWC: funding acquisition, methodology, project administration, resources, and supervision. JK, KWK, and YWC: investigation. All authors: writing review and editing and conceptualization. All authors contributed to the article and approved the submitted version.
